# Human Platelet Mitochondrial Function Reflects Systemic Mitochondrial Alterations: A Protocol for Application in Field Studies

**DOI:** 10.3390/cells10082088

**Published:** 2021-08-14

**Authors:** Florian Hoppel, Luiz Felipe Garcia-Souza, Wilhelm Kantner-Rumplmair, Martin Burtscher, Erich Gnaiger, Dominik Pesta, Elisa Calabria

**Affiliations:** 1 Oroboros Instruments, 6020 Innsbruck, Austria; flo.hoppel@gmail.com (F.H.); luiz.garcia@oroboros.at (L.F.G.-S.); erich.gnaiger@oroboros.at (E.G.); 2 Department of Sport Science, University of Innsbruck, 6020 Innsbruck, Austria; Martin.Burtscher@uibk.ac.at; 3 D. Swarovski Research Laboratory, Department of Visceral, Transplant Thoracic Surgery, Medical University of Innsbruck, 6020 Innsbruck, Austria; 4 Psychosomatic Pain Ambulance, University Hospital for Medical Psychology and Psychotherapy, 6020 Innsbruck, Austria; wilhelm.kantner-rumplmair@tirol-kliniken.at; 5 German Aerospace Center (DLR), Institute of Aerospace Medicine, 51147 Cologne, Germany; 6 Institute for Clinical Diabetology, German Diabetes Center, Leibniz Center for Diabetes Research at Heinrich Heine University, 40225 Düsseldorf, Germany; 7 German Center for Diabetes Research (DZD e.V.), 85764 München-Neuherberg, Germany; 8 Centre for Endocrinology, Diabetes and Preventive Medicine (CEDP), University Hospital Cologne, 50924 Cologne, Germany; 9 Cologne Excellence Cluster on Cellular Stress Responses in Aging-Associated Diseases (CECAD), 50931 Cologne, Germany; 10 Department of Neurosciences, Biomedicine and Movement Sciences, University of Verona, 37131 Verona, Italy

**Keywords:** platelets, respirometry, field study, physical exercise, quality control, mitochondrial function

## Abstract

Human blood cells may offer a minimally invasive strategy to study systemic alterations of mitochondrial function. Here we tested the reliability of a protocol designed to study mitochondrial respiratory control in human platelets (PLTs) in field studies, using high-resolution respirometry (HRR). Several factors may trigger PLT aggregation during the assay, altering the homogeneity of the cell suspension and distorting the number of cells added to the two chambers (A, B) of the Oroboros Oxygraph-2k (O2k). Thus, inter-chamber variability (∆*ab*) was calculated by normalizing oxygen consumption to chamber volume (*J*_*O*2_) or to a specific respiratory control state (flux control ratio, *FCR*) as a reliable parameter of experimental quality. The method’s reliability was tested by comparing the ∆*ab* of laboratory-performed experiments (LAB, *N* = 9) to those of an ultramarathon field study (three sampling time-points: before competition (PRE, *N* = 7), immediately after (POST, *N* = 10) and 24 h after competition (REC; *N* = 10)). Our results show that ∆*ab* *J*_*O*2_ changed PRE-POST, but also for LAB-POST and LAB-REC, while all ∆*ab FCR* remained unchanged. Thus, we conclude that our method is reliable for assessing PLT mitochondrial function in LAB and field studies and after systemic stress conditions.

## 1. Introduction

Investigating changes of mitochondrial function improves our understanding of the physiology and pathophysiology of various diseases [1,2], aging [3], obesity [4], or acclimatization to physical activity as opposed to a sedentary lifestyle [5,6]. High-Resolution Respirometry (HRR) or High-Resolution FluoRespirometry (HRFR) is widely used for analysis of mitochondrial oxidative phosphorylation (OXPHOS), using different sample preparations of intact or permeabilized cells, permeabilized muscle fibres, isolated mitochondria, and tissue homogenates [1,2]. Various combinations of mitochondrial coupling control states and electron transfer pathways can be investigated using Substrate-Uncoupler-Inhibitor-Titration (SUIT) protocols [1,2,7].

Changes in mitochondrial function in response to different exercise training interventions have been assessed in either isolated mitochondria or permeabilized muscle fibres obtained from vastus lateralis muscle biopsies [8]. However, results may vary depending on the SUIT protocol or tissue as well as the method of tissue preparation [1,2], the type of muscle and its fiber-type composition [9,10], the sampling depth [7] and mitochondrial subpopulations studied [9]. Human blood cells may offer a minimally invasive strategy to study the systemic alterations of mitochondrial function [1,11,12,13,14,15]. In primates and older adults, blood cell bioenergetic changes are associated with skeletal muscle and cardiac mitochondrial alterations [12,13].

Usually, HRR or HRFR are performed in a laboratory under controlled conditions. However, when studying e.g., physiological responses following endurance competitions, on-site setups are necessary to avoid prolonged sample transport, which could affect experimental results [11]. Time constrains due to multiple participants finishing within a short time period require fast and efficient collection, processing and analyses of the samples while still maintaining highest standards.

Both peripheral blood mononuclear cells (PBMCs) and PLTs are easily obtainable and suitable for HRR. Although purification procedures of both blood cell subpopulations from whole blood are similar, PLTs preparations are easier as they require less blood and fewer centrifugation steps [11]. In comparison, sample preparation for PBMCs takes about one hour, which is comparable to permeabilized muscle fibre preparations, while only 40 min are needed for PLT preparations [16]. Furthermore, PBMCs are composed of heterogeneous subpopulations of cells including monocytes, lymphocytes (T- and B- cells, natural killer cells), and dendritic cells [11,15]. These different cell types often exhibit specific respirometric profiles that are potentially differently affected by exercise [15]. As systemic stress such as prolonged exercise induces shifts within individual subgroups of PBMCs (decrease of lymphocyte but increase of monocyte populations) [17], potential changes in PBMC respiration following exercise could reflect either the change in the composition of PBMC subpopulations or changes in mitochondrial function.

Hence, metabolically highly active but homogenous PLTs might be a valuable surrogate to investigate systemic changes of mitochondrial function, since they are involved in a variety of systemic reactions (e.g., hemostasis, immunoregulation, inflammation or oxidative stress) [18,19,20]. Assessing PLT bioenergetics as a systemic marker has been performed in the context of various diseases [15,19,21,22,23,24,25,26,27,28] as well as training studies [29,30,31,32,33]. However, changes in PLT bioenergetics after an ultramarathon competition in the context of a field study has not been assessed so far.

Thus, we developed a simple and fast method for PLT isolation from blood samples and on-site analysis of mitochondrial function. The aim of this work was to assess methods’ reliability derived from a parameter of experimental quality obtained during field and laboratory experiments. Subsequently, we provide a detailed description of required materials, experimental procedures and workflow of sample preparation.

## 2. Materials and Methods: A Guideline for HRR in PLTs

This experimental approach was developed for an ultramarathon field study involving 10 male ultramarathon runners. PLT mitochondrial respiration was measured at 3 time-points: ~12 h before the start of the competition (PRE), within 10 min (POST) and 24 h (REC) after finishing the race. We evaluated our method by comparing flux control rations (*FCR*), a reliable parameter of experimental quality, obtained from field and laboratory experiments.

### 2.1. Sampling and Cell Purification

Our separation procedure offers a fast way to obtain a sufficient cell number suitable for HRR. Instead of using three consecutive centrifugation steps for PLTs purification, we use only two with the first centrifugation step being performed at slightly higher *g*, to ensure sedimentation of cells with higher mass, thus improving sample purity. Despite the higher loss of PLTs during this step, the Oroboros O2k is sensitive enough to obtain valid results from a limited number of cells recovered using our protocol [11,34]. Required materials and chemicals for sampling, purification, and performing SUIT protocols are given in Table 1, Table 2 and Table 3.

For this protocol, the time required for sampling and PLT purification and transfer of platelets into the O2k chambers is 40 min (Table 4). Based on our experience, PLT purification is recommended to be performed as quickly as possible without changing environmental temperature, since PLTs are very sensitive to these factors and could be activated to form aggregates.

Egtazic acid (EGTA) can be added to the sample to avoid aggregation [11]. In case of a delay, PRP can be temporarily stored at 37 °C and protected from light (warm bath in a closed polystyrene box) between the 2 centrifugation steps for up to 10 min. Storing the PRP for longer may increase the risk of PLT aggregation. In general, we observed O2k experimental flux being less effected by artefacts after storing PRP for a short time (~5 min) before pelleting the cells (2nd centrifugation step). Moreover, also storing the purified platelets following resuspension in mitochondrial respiration medium (MiR05, after the 2nd centrifugation step) should be avoided, since PLT O_2_ flux tends to be lower or even deteriorate after storage in MiR05. For all centrifugation steps, the temperature was set to 23 °C.

If a device for cell counting is available, each sample should be measured following resuspension in pre-heated (37 °C) MiR05 and diluted to a final cell concentration of 200 × 10^6^ cells per mL by adding MiR05. This concentration has been previously recommended by Sjövall et al. for maximal O_2_ flux in human PLTs (see steps 6, 8 and 9 of Table 4) [34]. At the end of HRR experiments, we recommend recovering subsamples from the O2k chamber and storing these on dry ice after for subsequent determination of protein concentration or citrate synthase activity (see step 12 of in Table 4).

### 2.2. High-Resolution Respirometry

The Oroboros O2k for High-Resolution FluoRespirometry (Oroboros Instruments, Innsbruck, Austria) was used to measure sample O_2_ consumption using two 2-mL Duran glass chambers under continuous stirring and temperature control at 37 °C ± 0.001 °C [1]. Since the volume-specific O_2_ flux at the experimental concentration of PLTs is low, rigorous corrections for oxygen background flux, two-point oxygen signal calibration, and an adequate number of data points in each respiratory state are required. Experimental background flux evaluation is usually performed after every O2k assembly or at regular intervals, respectively, to exclude instrumental artefacts [35]. Thus, this is an important part of quality control also in field studies and was performed right after O2k assembly at the beginning of our project prior to the first experiments. The background settings were further used for all 3 experimental days of the ultramarathon study. See Table A1 in the Appendix A for experimental background details.

The polarographic oxygen sensors (POS) were calibrated to ambient air O_2_ pressure at physiological temperature (37 °C) once every day with MiR05, which is commonly performed in the mitochondrial respiratory medium used for subsequent experiments as recommended (see Table 3 for details of mitochondrial respiration medium MiR05) [1,35,36,37]. The respiration media used for calibration was removed from the chambers by aspiration and the prepared samples were transferred into the O2k chamber. After closing both O2k chambers, the O_2_ flux signal (*J*_*O*2_; pmol/s/mL) stabilizes and the oxygen consumption in the different respiratory states can be recorded. Transitions from a state to another, as well as specific activation or inhibition of mitochondrial respiratory complexes were obtained by titration of chemicals according to the SUIT-protocol described below.

### 2.3. SUIT-Protocol for Platelets

To analyze mitochondrial function in PLTs, we used a modified Oroboros SUIT 8 protocol optimized for permeabilized cells [1]. Saturating, but not inhibiting substrate concentrations as well as physiological temperature during the experiment are important quality criteria for optimal O_2_ flux [37]. Hence, appropriate substrate concentrations were evaluated prior to the experiments and the experimental temperature was set to 37 °C.

The following respiratory states and electron transfer-pathways are characterized with SUIT-protocols: ROUTINE (R), LEAK (L), OXPHOS (P), ET (E), and ROX. ROUTINE reflects respiration in non-permeabilized cells where respiration is sustained by endogenous substrates (R) or by pyruvate and malate (R(PM)) sustaining the NADH-pathway (N). Following plasma membrane permeabilization with the mild detergent digitonin, LEAK (L) respiration is obtained (N(PM)*_L_*). The presence of the reducing substrates pyruvate and malate (PM) and the subsequent addition of ADP triggers the transition from the LEAK to the NADH-linked OXPHOS state (P) (N(PM)*_P_*, N-pathway in presence of PM and ADP). N-linked OXPHOS capacity is fully stimulated by addition of glutamate (G) (N(PGM)*_P_*). The synergy with complex II was evaluated following addition of succinate (S), (NS; titration of S to PGM (NS(PMG)*_P_*)), electron transfer-pathway capacity (ET) in non-coupled conditions is obtained by stepwise titrations of Carbonyl cyanide m-chloro phenyl hydrazine (CCCP) (NS(PGM)*_E_*). Selective inhibition of complex I with rotenone (Rot) allows evaluation of the contribution of the S-pathway to ET-capacity (S(Rot)*_E_*). Residual oxygen consumption (ROX) was obtained by adding Antimycin A, an inhibitor of complex III.

Volume-specific *J*_*O*2_ can be normalized per cell number (pmol/s/10^6^ cells per mL) when the cell concentration is known, or per specific amount of marker (protein content [pmol/s/mg)]) if citrate synthase activity or mitochondrial protein content are evaluated on subsamples of PLTs (see Table 4 for workflow). *FCR* are obtained by normalization of O_2_ fluxes to a reference state, normally the highest one observed during the SUIT protocol. Flux control efficiency (*j_Z-Y_*) expresses the relative change of respiration in a defined coupling state in a step, caused by a specific metabolic control variable. Hence, *j_Z-Y_* enables analyses of an O_2_ flux step, caused by the titration of a single substrate, by using the next higher flux as reference, while *FCR* express fluxes relative to a reference state (Table 5) [2,38]. Results expressed by *FCR* and *j_Z-Y_* are statistically more robust as they are independent of cell number or external mitochondrial markers [2]. However, they only express qualitative, and not quantitative mitochondrial respirometric changes [2]. *FCR* and *j_Z-Y_* investigated using our SUIT-protocol are given in Table 5. Raw data were corrected by ROX (non-respirometric oxidative side reactions) subtraction prior to calculation of both *FCR* and *j_Z-Y_*.

**Figure 1:**
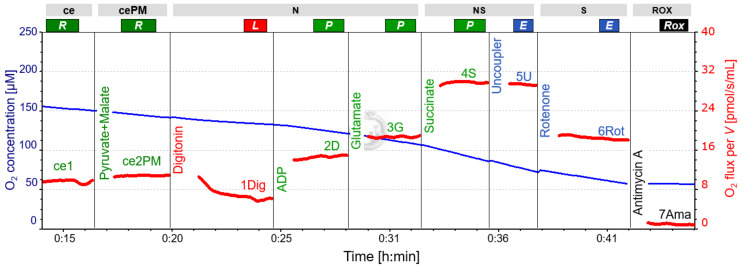
Representative respirometric trace following SUIT 8 protocol. Blue line: O_2_ concentration, measured at 2-second time intervals, showing the decline over time and the effect of oxygen added with titrations (particularly with substances dissolved in ethanol containing a high concentration of O_2_; see CCCP and rotenone titrations). Oxygen concentration was sufficient to maintain cell respiration. Red line: volume-specific O_2_ flux, calculated as the negative time derivative of O_2_ concentration, corrected for instrumental background, and expressed as pmol/s/mL. The slope was calculated for each data point using a non-linear fit over the preceding 25 data points. Titrations cause intermittent disturbances of the slope due to O_2_ injections, and these sections were removed. A slight but constant decrease in O_2_ flux from NS(PGM)*_P_* to NS(PGM)*_E_* and S(Rot)*_E_* is apparent. For titration steps see Table 3. The representative experiment was performed in LAB conditions. Abbreviations: ce, presence of the sample (cells); cePM, presence of the sample, pyruvate and malate; N, N-pathway: NADH electron transfer-pathway state; NS, NS-pathway: CI&CII linked electron transfer-pathway state; S, S-pathway: succinate-linked (CII-linked) electron transfer-pathway state; ROX, Residual oxygen consumption; R, Routine state; L, Leak state; P, Oxidative Phosphorylation (OXPHOS); E, Electron transfer pathway (ET); ADP, adenosine di-phosphate.

**Figure 2:**
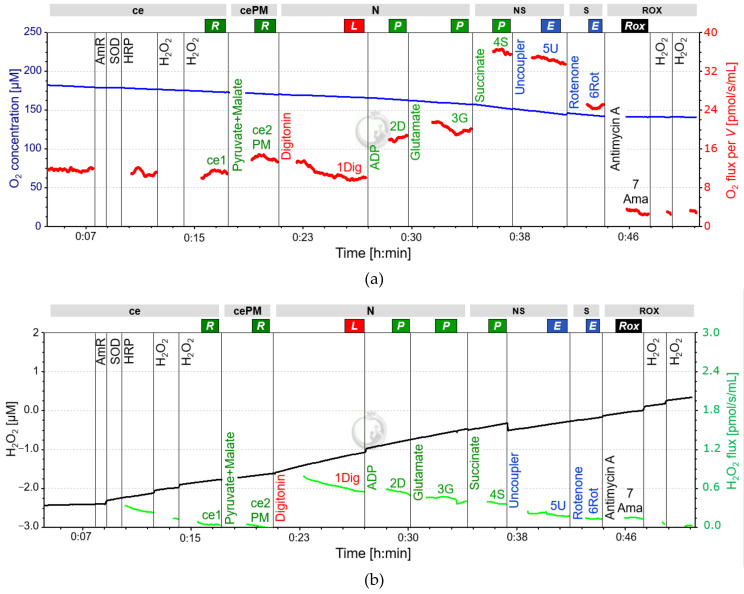
Representative fluo-respirometric trace following SUIT 8 protocol. (**a**): The blue line shows the O_2_ concentration [µM] measured at 2-second time intervals, the red line represents the volume-specific O_2_ flux [pmol/s/mL] as described in Figure 1. The volume-specific flux shows instability in relation to the experiment of Figure 1, starting at the point of the first chemical titrations. The decrease in O_2_ flux from NS(PGM)*_P_* to NS(PGM)*_E_* is intensified compared to Figure 1. The time interval between titration of ADP and G was too short to determine N(PM)*_P_*. (**b**): The black line is the amperometric signal, converted from the raw data of fluorescence to the total concentration of AmR [µM] after calibration with H_2_O_2_. The amperometric signal is characterized by an average increase over time with slight stepwise changes induced by titrations. The green line represents the calculated H_2_O_2_ flux per volume [pmol/s/mL]. The digitonin titration causes a burst in H_2_O_2_ release, slowly decreasing over time, but masking possible changes in H_2_O_2_ release. The representative experiment was performed in the laboratory of Oroboros Instruments. Abbreviations: ce, presence of the sample (cells); cePM, presence of the sample, pyruvate and malate; N, N-pathway: NADH electron transfer-pathway state; NS, NS-pathway: CI&CII linked electron transfer-pathway state; S, S-pathway: succinate-linked (CII-linked) electron transfer-pathway state; ROX, Residual oxygen consumption; R, Routine state; L, Leak state; P, Oxidative Phosphorylation (OXPHOS); E, Electron transfer pathway (ET); ADP, adenosine di-phosphate; AmR, Amplex UltraRed; SOD, Superoxide Dismutase; HRP, Horseradish Peroxidase; H_2_O_2_, Hydrogen Peroxide.

Usually, E is equal to or greater than P, thus the ratio of E to P after uncoupler titration is E ≥ P [2]. OXPHOS exceeding ET (E < P) is theoretically impossible but can be caused e.g. by low O_2_ concentration in the O2k chambers inhibiting respiration [2]. In some runs, we observed E < P (<5%), despite sufficient oxygen (see Figure 1 and Figure 2). The reason for this observation remains unclear but is likely an experimental artefact. For this reason, we assumed E = P in our experiments [2]. Hence, NS(PGM)*_P_* is the more representative flux state for normalization in calculating all *FCR* and the *J_Z-Y_* for Rotenone [1-S(ROT)*_E_*/NS(PGM)*_P_*].

Reactive oxygen species (ROS) as a by-product of mitochondrial respiration are known to increase in response to exercise [39]. ROS can be assessed in parallel with respiration in the O2k by using the optional O2k-Fluorescence LED2-module and addition of Amplex UltraRed (AmR), Horseradish peroxidase (HRP), and superoxide dismutase (SOD) into the medium prior to the SUIT-protocol (High-Resolution FluoRespirometry; HRFR) [40]. Since mitochondrial electron transfer complexes I and III are considered the main sources of ROS and ROS formation is further associated with changes in intrinsic uncoupling (increased LEAK-respiration), the real-time determination of the amperometric slope in N(PM)*_L_*, N(PGM)*_P_*, NS(PGM)*_P_* and S(Rot)*_E_* is of particular interest [2,40].

When assessing ROS in our experiments, we observed a distinct and abrupt increase of the amperometric slope (“oxidative burst”) following digitonin titration for permeabilization. Despite a gradual decline of the subsequent signal, possible fluorometric changes induced by substrate addition were overlapped by this oxidative burst and could therefore not be evaluated. Hence, it is not recommended to use the LED2-module in this protocol for human PLTs, since a meaningful analysis of the amperometric slope is not possible in R state (see Figure 2). Calibration of the raw signal for fluorescence using H_2_O_2_ titrations to obtain the corrected AmR signal has to be performed both before and after the experimental SUIT protocol by [40]. See Table 3 and Table 4 for required materials and titration steps in addition to those used for HRR if the LED-2 module is also used.

## 3. Experimental Reproducibility: From Lab to Field

Despite using the same method for blood sampling, cell purification, and experimental SUIT protocol, the number of confounders is higher field studies compared to laboratory conditions. Likewise, excessive stress due to exercise-induced PLT activation can also contribute to PLT aggregation [41]. Cellular aggregation after resuspension of the pellet (after the 2nd centrifugation step) could worsen reproducibility: if a slightly different number of cells is added from the same sample to the two O2k chambers, it will result in different O_2_ flux rates. This effect might become more apparent when sample preparations are performed in field conditions or after strenuous exercise. To determine the reliability of our method, we compared data obtained from experiments performed both under standardized laboratory conditions and in the field using identical methods for sampling, sample preparation and HRR.

### 3.1. Methods and Study Design

Experimental reproducibility was evaluated by comparing O2k chamber variances of every experiment, where the sample obtained from each preparation procedure was divided into both O2k chambers (A and B) for HRR. Chamber variances (∆*ab*) were calculated by normalizing respiratory chamber differences (A−B) by the respective chamber averages, ∆*ab* = [(A − B)/AVG(A,B)], for both ROX non-corrected volume-specific O_2_ flux (*J*_*O*2_) and *FCR*. ∆*ab* as a technical parameter reflects the accuracy of the experimental procedure but is independent from any mitochondrial respirometric changes. All volume-specific fluxes (including ROX) and *FCR* collected by the present SUIT Protocol (Table 5) were included in the evaluation. During ideal experimental conditions and accurate sample handling, ∆*ab* is estimated to be zero at each respiratory state, considering that the two chambers contain the same number of cells and that the same amount of each compound is added in the two chambers. Since ∆*ab* can vary between −2 and +2 (depending on whether O_2_ flux is A > B or A < B), we used absolute values of variances for further statistical analyses (│∆*ab*│). Significant differences of │∆*ab*│ indicate cellular aggregations at least in one O2k chamber, thus a qualitative change in PLT sample preparation of experimental performance.

Two groups of participants were included in the evaluation: group 1 was sampled and assessed in field conditions, whereas for group 2, experiments were performed in the laboratory of Oroboros Instruments (LAB). Group 1 was composed of 10 participants of an ultramarathon project (age median: 51.5 years, range: 26–45 years; regular training/week: 8.5 h, range: 4–15 h), who were sampled three times (PRE-POST-REC) as described above [18]. Participants of group 2 were 9 recreational endurance athletes (running, cycling; age median: 31 years, range 29–41 years) who were sampled at least 24 h after their last training session.

**Figure 3:**
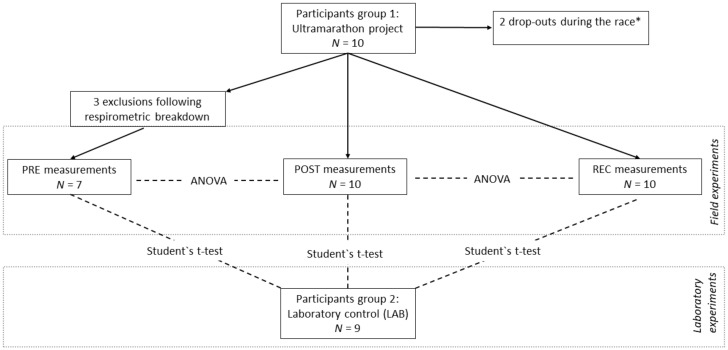
Study design and statistical approach. PRE, prerace measurements; POST, postrace measurements; REC, recovery measurements 24 h after individual finish; LAB, experiments performed in the laboratory of Oroboros Instruments. Repeated measures ANOVA with Bonferroni post-hoc tests was used for PRE-POST-REC comparison, highlighting the impact of systemic stress on experimental quality. Student’s t-test was used to compare LAB-PRE, LAB-POST, and LAB-REC, highlighting the impact of changed environmental conditions on experimental quality. │∆*ab*│ of all volume-specific fluxes (*J*_*O*2_) and *FCR* were used as parameters. * Experimental data from drop-outs are excluded in the evaluation of biological adaptations, but are included in the present statistics for experimental quality control.

Whole blood was taken from the median cubital vein (9 mL K3 EDTA, BD Vacutainer, BD diagnostics, New Jersey, United State. │∆*ab*│ calculated for all *J*_*O*2_ and *FCR* were compared as follows: LAB (*N* = 9) was compared to all field experiments (LAB-PRE, LAB-POST, LAB-REC; *N* = 10) to assess the method’s reliability in less controlled ambient conditions and the results obtained from the ultramarathon (PRE-POST-REC) were compared to evaluate the reliability under systemic stress conditions. The data of participants 2, 6, and 8 in PRE were excluded due to experimental problems (deteriorated experimental O_2_ flux), which reduced the sample of PRE (*N* = 7). These data are not shown in graphs and excluded from statistical analysis. All participants were informed about the procedure and gave written consent. The study was approved by the local ethics review board (University of Innsbruck, Institute of Sport Science). The study design and the statistical approach are shown in Figure 3.

### 3.2. Statistics

**Figure 4:**
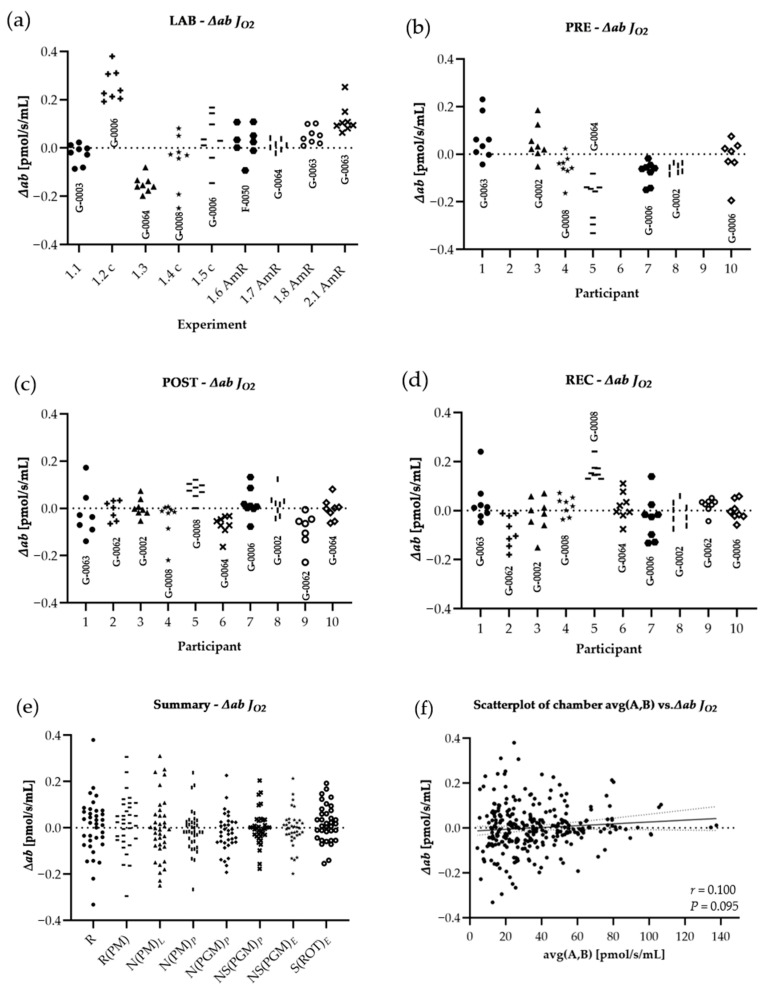
Distribution of variances for *J*_*O*2_: The difference of both chambers A and B, divided by the respective chamber average [∆*ab* = (A − B)/AVG(A,B)], calculated from ROX non-corrected data. Graphs show ∆*ab* from experiments performed in the laboratory of Oroboros Instruments (**a**) in field conditions before the ultramarathon race (**b**), right after (**c**) and 24 h after the finish (**d**). ∆*ab* for *J*_*O*2_ of all experiments (LAB, PRE, POST, REC) are shown in (**e**). ∆*ab* for *J*_*O*2_ are plotted against average chamber *J*_*O*2_ [pmol/s/mL] to evaluate the dependence of ∆*ab* on *J*_*O*2_ (**f**). The linear trend line and a 95% confidence interval are shown. See Table A1 in the appendix for O2k background correction values and Table A2 for O_2_ calibration of each experiment.

**Figure 5:**
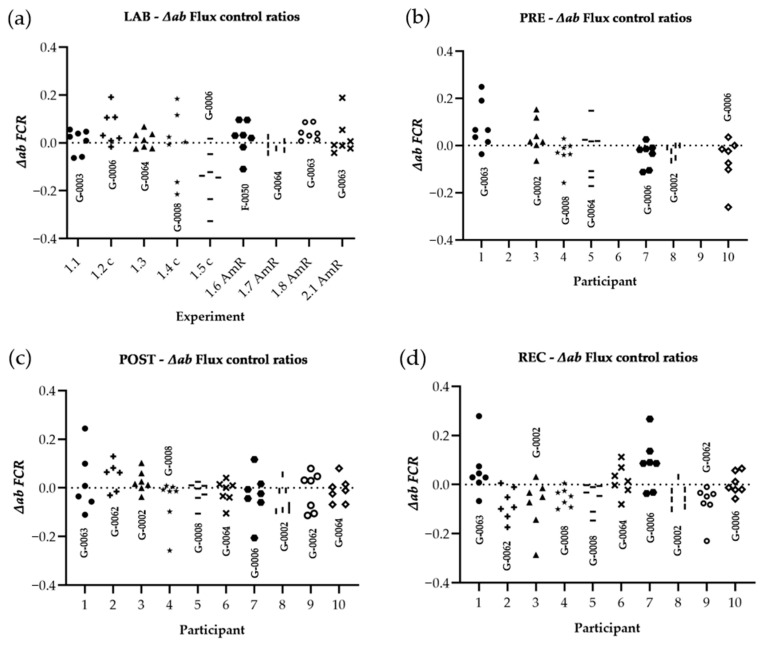

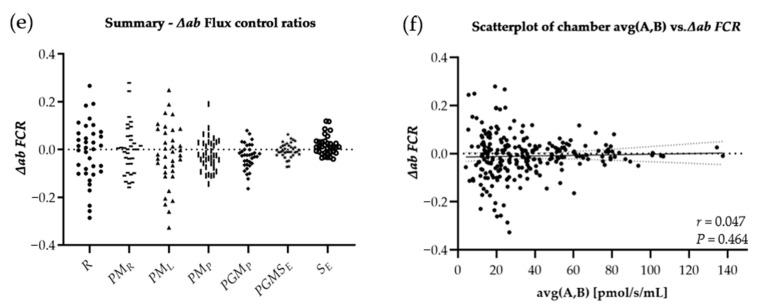
Distribution of variances for *FCR*: The difference of both chambers A and B, divided by the respective chamber average [∆*ab* = (A − B)/AVG(A,B)]. Raw data were corrected for ROX and *FCR* was calculated by normalizing for NS(PGM)*_P_* in every single experiment. Graphs show ∆*ab* of experiments performed in the laboratory of Oroboros Instruments (**a**), in field conditions before the ultramarathon race (**b**), right after (**c**), and 24 h after individual finish (**d**). ∆*ab* for *FCR* of all experiments (LAB, PRE, POST, REC) are shown in (**e**). ∆*ab* for *FCR* is plotted against average chamber *J*_*O*2_ [pmol/s/mL] to evaluate the dependence of ∆*ab* of *FCR* on *J*_*O*2_ (**f**). The linear trend line and a 95% confidence interval are shown. See Table A1 in the appendix for O2k background correction values and Table A2 for O_2_ calibration of each experiment.

All ∆*ab* of *J*_*O*2_ and *FCR* are shown for every single experiment performed in LAB conditions (*panel a* of Figure 4 and Figure 5), PRE (*panel b* of Figure 4 and Figure 5), POST (*panel c* of Figure 4 and Figure 5), and REC (*panel d* of Figure 4 and Figure 5). To evaluate whether ∆*ab* is dependent on the current rate of oxygen consumption averaged for both chambers (*J*_*O*2_), Spearman’s rank correlation was used (*panel f* of Figure 4 and Figure 5). To analyze changes LAB-PRE, LAB-POST, and LAB-REC, the Student’s t-test for unpaired samples was used for normally distributed data or the Mann-Whitney-U-Test for data not meeting the test requirements. To analyze PRE-POST-REC changes, a repeated one-way analysis of variance (ANOVA) with a post-hoc analysis (Bonferroni correction) was used, or the Wilcoxon signed-rank test and Friedman test. Absolute values of ∆*ab* were used for the ANOVA, t-test, and Mann-Whitney-U test (│∆*ab*│). A *p*-value below 0.05 was considered as statistically significant.

### 3.3. Results

Results are shown in Table 6. Regarding LAB and field experiments, the comparison of │∆*ab*│ for raw data showed significant changes only for │∆*ab*│N(PM)*_L_* LAB-POST (−62.1%) and │∆*ab*│S(ROT)*_E_* LAB-REC (−60.0%), and for the comparison of field experiments (PRE, POST and REC) only │∆*ab*│ N(PM)*_L_* (−99.6%) and │∆*ab*│ NS(PGM)*_E_* (−99.3%) were significantly different, both PRE-POST. All │∆*ab*│ *FCR* remained unchanged. ∆*ab* was not correlated to averaged chamber *J*_*O*2_, neither for ∆*ab* *J*_*O*2_ (*r* = 0.100, *p* = 0.095), nor for ∆*ab FCR* (*r* = 0.047, *p* = 0.464).

## 4. Discussion

This study evaluated the reliability of a method to assess platelet mitochondrial function by HRFR in the context of a field study. We compared a technical parameter independent of biological changes (∆*ab*), derived from HRFR experiments acquired during both laboratory and field conditions. We found slight differences in chamber variability│∆*ab*│, but not for derived │∆*ab*│ *FCR*. The observed difference of raw data does not reflect variations of experimental quality and was not associated with the level of oxygen consumption (*J*_*O*2_), but may rather be related to the operator’s level of experience. These findings indicate that the method is suitable for field studies, since the degree of variability observed in LAB and field conditions was similar.

Most previous studies using PLTs as a systemic respirometric marker involved different pathologies assessed in laboratory settings [15,21,22,27,28]. Even though de Lucas et al. used an O2k to assess HRR of human PLTs after ultra-endurance exercise, they did not evaluate various combinations of mitochondrial coupling control states and electron transfer pathway states [30]. Moreover, detailed information on the separation process and the ambient conditions during the experiments were not reported [30]. Previously, we published an extensive HRFR protocol performed in the context of a field study [18]. Even though we could not determine distinct race-related changes in mitochondrial function, the association of PLT respiration with individual race performance, oxidative stress, muscle damage and renal dysfunction indicates the involvement of blood cells in governing systemic processes [18]. If not, different methods can be used to assess blood-based bioenergetic changes, which are in part complementary, with each having their own strengths and limitations [15,42].

Assessing chamber variability (∆*ab*) for both *J*_*O*2_ and *FCR* allows for testing the reliability of a method and highlights how cellular aggregations may affect O_2_ consumption in both chambers. ∆*ab* is a technical parameter not affected by biological variability, reflecting quality of sample preparation, precision of titrations and experimental performance. LAB-to-field study conditions and PRE-POST comparison reveal differences of│∆*ab*│ regarding *J*_*O*2_ but not for *FCR*. However, it is expected that PLT aggregation during the experimental process would affect all mitochondrial respiratory states, resulting in change of ∆*ab* at all stages of the experiment. Hence, although several studies reported increased platelet aggregation in response to exercise [43], we suggest that neither varied experimental conditions (LAB-PRE), nor systemic stress after strenuous workloads (PRE-POST-REC) had an influence on data reproducibility.

However, differences in chamber variability could possibly be related to the operator’s experience, such as precision of titrations and/or timing of SUIT protocol injections. Other specific confounding variables relevant for the assay include the time for O_2_ flux to stabilize after digitonin titration. Presumably, the time for *J*_*O*2_ stabilization after full plasma membrane permeabilization, is not identical in both chambers. Hence, estimating the correct timepoint when *J*_*O*2_ is stable is difficult and depends on the operator’s decision. In general, the observation of chamber variability in only four tests out of 54 performed cannot be explained by PLT aggregation in the O2k chambers during the experiment. Additionally, │∆*ab*│ of *FCR* remained unchanged. Considering that *FCR*, but not ROX non-corrected *J*_*O*2_ are commonly used for further analysis, these differences are negligible. Overall, the technical validity and the influence of the operator’s experience on outcomes qualify this method as reliable for use in-field and laboratory studies.

PLT respiration turned out to be sensitive to prolonged storage of the sample before O2k experimental analysis. While 5 min of light-protected storage of PRP at physiological temperature seemed to be the optimum, we found 10 min to be the absolute maximum for stable O_2_ flux analysis. When downstream assays are delayed, a good alternative in field studies is storing the whole blood at both room temperature and 4 °C before the first centrifugation, which did not affect mitochondrial function up to 24 h [34]. However, in field studies, storage at 4 °C may not be possible and storage of up to 24 h may not be sufficient for transport to a laboratory and further analysis. Hence, any experimental procedure on-site using a field laboratory has to be planned carefully, avoiding any potential delays as much as possible. As potential mitochondrial recovery processes after exercise remain unclear, sampling right after exercise is recommended if the focus lies on acute adaptations. Hence, a delay in sampling, as well as a delay in sample preparation could negatively impact results. However, normalized chamber variances of all experiments were within the same range (−0.4 < ∆*ab* < 0.4), with a strong trend of LAB experiments being slightly more variable compared to field experiments (*p* = 0.05). Alternatively to handling of PLTs, adding the EGTA-chelating agent can prevent PLTs from forming aggregations [11]. However, an effect of this agent on respirometric outcomes cannot be excluded.

Substrates concentrations yielding maximal O_2_ flux are a prerequisite to obtain valid results [1]. HRR experiments performed with too low or too high substrate concentrations can result in impaired mitochondrial respiration [1]. The same applies to digitonin concentrations, which vary in PLTs compared to standard recommendations used for cells (PLTs: 1 μg/10^6^ cells, which is 200 µg/mL for a concentration of 200 × 10^6^ cells per mL) [1,34]. Using an optimal digitonin concentration, the plasma membrane is permeabilized following interaction with its high cholesterol content. Consequently, respiration remains constant over a defined range of digitonin concentrations by the free exchange of substrates between the cytosol and the immediate cell environment but tends to decline when the outer mitochondrial membrane with a low-cholesterol content is affected by an excess of digitonin [1,2]. The optimal digitonin concentration is commonly tested experimentally by stepwise titration of low digitonin concentrations (i.e., 10 μg/mL) until the maximal respiratory response is achieved, confirmed by subsequent outer mitochondrial membrane integrity test, by adding 10 μM cytochrome *c* [1,2].

Despite a high digitonin concentration, the final concentration per cell (f.c. 10 µg per 10^6^ cells) is low compared to standard recommendations. The reduced plasma membrane surface of PLTs, with small diameters of 1–3 µM, would require lower concentrations per cell, whereas the number of cells present in the chamber is much higher compared to other cell types [11]. Thus, high final concentrations may contribute to the observed oxidative burst when using the LED2-Module for fluorometry. In pilot studies, we found that the outer mitochondrial membrane remained intact, using the cytochrome c test, with a final digitonin concentration of 200 µg/mL, confirming recommendations of Sjövall et al. [34]. However, recent findings from our working group suggest that a sufficient O_2_ flux can be obtained using lower concentrations of digitonin at similar cell concentrations, but by using an adapted SUIT protocol. Thus, further studies of protocol development can reduce the risk of using too high digitonin concentrations by stepwise titrations until optimal O_2_-flux is reached.

EDTA is recommended as an anticoagulant for PLT respirometry, ensuring the best experimental results and prevention of platelet activation compared to heparin, citrate, and acid citrate dextrose [34]. However, we also found sodium citrate to be used in one study [23]. To our knowledge, it has not been tested explicitly, whether EDTA is the optimal anticoagulant for PLT respirometry in general or if this is dependent on the SUIT protocol. Hence, different anticoagulants should be tested together with different SUIT protocols for human PLTs.

## 5. Conclusions

In conclusion, the method presented here was optimized to investigate PLT mitochondrial qualitative function in a time- and resource-efficient manner. The method was found to yield reliable results in both LAB and field conditions, as well as after systemic stress. Experienced operators and swift performance of all the steps can prevent aggregation of platelets, which can further improve outcomes. ∆*ab* is a useful parameter to assess the reliability of HRR experiments in field and laboratory settings. It reflects both the homogeneity of the sample and the precision of the operator. In order to exactly identify and select the phase of O_2_ flux stabilization after single titration steps, it is necessary to define a minimum time interval and/or an optimal number of recordings, which requires a high level of user experience.

## Figures and Tables

**Table 1:** Materials for blood sampling, O2k sample preparation, and subsample storage.

	Materials for Blood Sampling	Provider
1	9 mL VACUETTE^®^ K3EDTA tubes and 21-gauge butterfly needles (2 per participant)	Greiner Bio-One, Kremsmünster, Austria
2	Set of automatic pipettes and tips (1 × 2.5 mL, 1 × 1 mL, 1 × 2 µL)	
3	Respiratory medium (MiR05) ^1^ (minimum 4.5 mL for each experiment; SUIT, background exp., O_2_ calib.)	Oroboros Instruments, Innsbruck, Austria
4	Centrifuge with a swinging bucket rotor and slots for 50 mL and 15 mL tubes and 9 mL vials; min. 1000× *g* available	Hettich Rotina 35, Tuttlingen, Germany
5	Eppendorf centrifuge with slots for 1.5 mL tubes; min. 1000× *g* available	Eppendorf AG, Hamburg, Germany
6	Eppendorf tubes, 1.5 mL (2 tubes for each SUIT experiment)	Eppendorf AG, Hamburg, Germany
7	Thermometer	
8	50 mL Falcon tubes (2 for each sampling/experiment)	BD Becton Dickinson GmbH, Heidelberg, Germany
9	Polystyrene box with dry ice, protected from light	
10	PBS (1 mL for each experiment)	BE 17-516F, Lonza
11	Automated Cell counter	See discussion

^1^ Additional respiration medium is needed for diluting cell concentrations to 200 × 10^6^ cells per mL and for storing subsamples for measurement of additional parameters (see Table 4).

**Table 2:** Materials for O2k FluoRespirometry (HRFR).

	Materials for Respirometry	Provider
1	O2k for high-resolution Fluo-respirometry including Titration-Injection micropump (TIP), ISS-Integrated Suction System (ISS) (6 instruments)	Oroboros Instruments, Innsbruck, Austria
2	Aqua distilled; 70% Ethanol; 100% Ethanol	
3	Respiratory medium (MiR05) (4.5 mL for both each O_2_ calibration and instrumental O_2_ background exp.)	Oroboros Instruments, Innsbruck, Austria
4	Chemicals for O2k assay (see Table 4)	
5	O2k titration sets (6 × 10 µL, 6 × 25 µL, 1 × 50 µL, 1 × 100 µL, 1 × 500 µL; Hamilton Syringes) (number according to number of simultaneous working researchers)	Oroboros Instruments, Innsbruck, Austria
6	O2k-Fluo LED2-Module (6 modules) ^1^	Oroboros Instruments, Innsbruck, Austria
7	O2k titration set (3 × 10 µL; Hamilton syringes) (number according to number of simultaneous working researchers)	Oroboros Instruments, Innsbruck, Austria
8	Chemicals for fluorometry (see Table 4)	

^1^ At least 6 instruments are recommended for a study design used in Hoppel et al (2021) [18].

**Table 3:** Chemicals and SUIT-protocol for O2k FluoRespirometry with LED-2-modules based on the experimental SUIT 8 protocol.

Step	Chemical	Stock Concentration	Titration Volume	Final Concentration
*SUIT protocol for HRR*				
ce1	Sample			
ce1P	Pyruvate	2 M	5 µL	5 mM
ce2M	Malate	400 mM	10 µL	2 mM
1Dig	Digitonin	50 mg/mL	8 µL	200 µg/mL
2D	Adenosine diphosphate	500 mM	4 µL	1 mM
3G	Glutamate	2 M	5 µL	5 mM
4S	Succinate	1 M	20 µL	10 mM
5U ^1^	Carbonyl cyanide m-chloro phenyl hydrazine	1 nM	1 µL/step	0.5 µM steps
6Rot	Rotenone	1 mM	4 µL	2 µM
7Ama	Antimycin A	5 mM	0.5 µL	1.25 µM
*Optional SUIT protocol for Fluorometry*				
ce1AmR	Amplex UltraRed	10 mM	1 µL	5 µM
ce2HRP	Horseradish Peroxidase	500 U/mL	4 µL	1 U/mL
ce3SOD	Superoxide Dismutase	5000 U/mL	1 µL	2.5 U/mL
perform SUIT protocol for respirometry ^2^				
ce4H_2_O_2_	Hydrogen Peroxide	40 µM	5 µL	0.1 µM
ce5H_2_O_2_	Hydrogen Peroxide	40 µM	5 µL	0.1 µM

Additional chemicals are required: Mitochondrial respiration medium MiR05 according to Fasching, et al [35] containing EGTA (0.5 mM), MgCl2. 6 H2O (2 mM), Lactobionic Acid (60 mM), Taurine (20 mM), KH2PO4 (10 mM), HEPES (20 mM), D-Sucrose (110 mM), BSA (1 g/L) [22]; Dithionite solution (30 mM) in Phosphate buffer (50 mM, pH 8) for O_2_ background calibration. ^1^ Multiple titrations to reach maximum flux of ET capacity (NS(PGM)*_E_*); no uncoupling effect could be observed in our experiments. SUIT-protocol will be discussed according to [1]. ^2^ SUIT-protocol for respirometry has to be performed after ce3SOD, followed by ce4H_2_O_2_.

**Table 4:** Protocol for sample and simultaneous O2k preparation.

Contents of Every Single Step of the Procedure	
1.	Pre-heat MiR05 before use in a prepared water bath at 37 °C in a Styrofoam box shielded from light.
2.	Run air calibration of the O2k with MiR05 at 37 °C including a stirrer test according to Fasching & Gnaiger, 2018 [36]; keep them running in MiR05 until cells are added.
3.	Collect 2 × 9 mL blood in VACUETTE^®^ K3EDTA vials from each participant
5.	Centrifuge both 9 mL K3EDTA vials at 400× *g* at RT for 10 min; set no brake to avoid perturbation of cell layers.
6.	Pipette PRP gently until ~1 cm above cell pellet remains, transfer PRP into a 50 mL Falcon tube, store regularly for up to 10 min at 37 °C protected from light.
7.	Centrifuge PRP to collect PLTs and PPP (1000× *g*, RT, 10 min, acceleration 9, brake 6).
9.	Resuspend cell pellet (step 7) by gentle pipetting in 4.5 mL pre-heated MiR05; use 1 mL tips.
10.	Determine cell concentration ^1^ and dilute to 200 × 10^6^ cells per mL cells with preheated MiR05.
11.	Siphon off all MiR05 from the O2k and add 2.25 mL sample to each O2k chamber, close the chambers avoiding air bubbles, and siphon off excess medium from the stopper receptacle.
12.	Start data recording with DatLab.
13.	After terminating the respirometric experiment, transfer subsamples of the cells suspension (1 mL) from the O2k chamber while the stirrer is running, pellet the cells in Eppendorf tubes (1000× *g*, RT, 10 min, brake), resuspend in 300 µL PBS, and store on dry ice in Eppendorf tubes.

^1^ The preferred instrument is the Sysmex cell counter. See discussion for recent advancements. PRP, platelet rich plasma.

**Table 5:** Respiratory states, flux control ratios, and flux control efficiency, ROX-corrected.

State	Flux	After Event	*FCR*	*J_Z-Y_*
ROUTINE	R	ce1	*R*	
ROUTINE (PM)	R(PM)	ce2PM	*PM_R_*	
LEAK (L)	N(PM)*_L_*	1Dig	*PM_L_*	
OXPHOS (P)	N(PM)*_P_*	2D	*PM_P_*	1-N(PM)*_L_*/N(PM)*_P_*
OXPHOS (P)	N(PGM)*_P_*	3G	*PGM_P_*	1-N(PM)*_P_*/N(PGM)*_P_*
OXPHOS (P)	NS(PGM)*_P_*^1^	4S		1-N(PGM)*_P_*/NS(PGM)*_P_*
ET (E)	NS(PGM)*_E_*	5U	*PGMS_E_*	1-NS(PGM)*_E_*/N(PGMS)*_p_*
ET S (E)	S(Rot)*_E_*	6Rot	*S_E_*	1-S(Rot)*_E_*/NS(PGM)*_P_*^2^

ROUTINE (R) respiration of intact cells with endogenous substrates; LEAK (L) in the absence of adenylates; OXPHOS (P) in the presence of saturating [ADP]; ET (E) after uncoupler titration to obtain maximum flux. ^1^ NS(PGM)_P_ was used for normalization for calculating FCR. ^2^ NS(PGM)_P_ instead of NS(PGM)*_E_* was used for normalization.

**Table 6:** Results of │∆*ab*│ of *J*_*O*2_ and *FCR*.

		Field			
	LAB	PRE	POST	REC	Adjusted *p*
│∆*ab*│ Volume specific flux					
R	0.099 ± 0.112	0.066 ± 0.103	0.075 ± 0.058	0.095 ± 0.057	0.983
R(PM)	0.097 ± 0.095	0.064 ± 0.096	0.059 ± 0.058	0.080 ± 0.067	0.983
N(PM)*_L_*	0.149 ± 0.106 *	0.090 ± 0.091 ^$^	0.057 ± 0.069	0.081 ± 0.072	0.102
N(PM)*_P_*	0.076 ± 0.073	0.068 ± 0.089	0.041 ± 0.043	0.052 ± 0.053	0.204
N(PGM)*_P_*	0.093 ± 0.077	0.045 ± 0.044	0.045 ± 0.049	0.054 ± 0.046	0.135
NS(PGM)*_P_*	0.079 ± 0.079	0.034 ± 0.048	0.036 ± 0.033	0.040 ± 0.048	0.905
NS(PGM)*_E_*	0.080 ± 0.078	0.038 ± 0.044 ^$^	0.040 ± 0.047	0.043 ± 0.051	0.066
S(ROT)*_E_*	0.083 ± 0.071 ^#^	0.053 ± 0.052	0.059 ± 0.040	0.032 ± 0.034	0.513
ROX	0.245 ± 0.174	0.311 ± 0.303	0.401 ± 0.429	0.277 ± 0.204	0.565
│∆*ab*│ Flux control ratio					
*R*	0.095 ± 0.084	0.049 ± 0.060	0.079 ± 0.076	0.136 ± 0.078	0.298
*PM_R_*	0.066 ± 0.053	0.038 ± 0.059	0.062 ± 0.075	0.094 ± 0.081	0.479
*PM_L_*	0.124 ± 0.101	0.096 ± 0.104	0.066 ± 0.061	0.086 ± 0.068	0.368
*PM_P_*	0.056 ± 0.043	0.050 ± 0.061	0.060 ± 0.034	0.045 ± 0.031	0.565
*PGM_P_*	0.057 ± 0.052	0.021 ± 0.026	0.043 ± 0.039	0.049 ± 0.026	0.576
*PGMS_E_*	0.020 ± 0.015	0.013 ± 0.013	0.026 ± 0.024	0.029 ± 0.022	0.060
*S_E_*	0.019 ± 0.015	0.030 ± 0.038	0.035 ± 0.036	0.029 ± 0.024	0.607

Data are shown as mean ± SD of differences between O2k chambers A and B, normalized by the chamber average [|(A − B)|/(avg(A,B)] of *J_O2_* and *FCR*. Adjusted *p*-values of ANOVA with Bonferroni post-hoc for analysis of the field experiments (PRE-POST-REC) are given (right column). A t-test for independent samples was used for LAB-PRE analyses (*p*-values not shown). * significance between LAB-POST (unpaired t-test); *p* = 0.05. ^#^ significance between LAB-REC (unpaired *t*-test); *p* = 0.05. ^$^ significance between PRE-POST (ANOVA with post-hoc test); *p* = 0.05.

## Data Availability

The raw data supporting the conclusions of this article will be made available by the authors, without undue reservation.

## Appendix A

**Table A1:** O2k background O_2_ flux in LAB and ultramarathon experiments. Values are shown in [pmol/s/mL].

	O_2_ Background a°		O_2_ Background b°	
O2k Serial Number	A	B	A	B
LAB				
G-0003	−2.8046	−2.0394	0.0287	0.0275
G-0006	−2.2446	−2.9382	0.0295	0.0311
G-0064	−2.0428	−1.8178	0.0249	0.0273
G-0008	−2.3598	−2.7908	0.0301	0.0334
G-0006	−2.2446	−2.9382	0.0295	0.0311
F-0050 *	−2.0000	−2.0000	0.0250	0.0250
G-0064	−2.4825	−2.0258	0.0305	0.0275
G-0063	−1.6941	−2.2292	0.0273	0.0296
G-0063	−2.4168	−2.4971	0.0327	0.0317
PRE, POST, REC				
G-0002	−2.5226	−3.0570	0.0289	0.0313
G-0006	−2.0208	−1.7848	0.0318	0.0309
G-0008	−2.7778	−2.1008	0.0263	0.0263
G-0062	−2.0303	−1.5078	0.0267	0.0250
G-0063	−1.6941	−2.2292	0.0273	0.0296
G-0064	−2.4825	−2.0258	0.0305	0.0275

O2k background correction for all used O2k in LAB and ultramarathon experiments. Experimental background for the ultramarathon experiments were performed once on the day before PRE experiments, and used PRE, POST, and REC. Used LAB experiments were performed in a time range of 2 months, where O2k were disassembled and repeatedly background-corrected meanwhile. * default background settings were used. O_2_ background a°: Intercept, flux at zero oxygen. O_2_ background b°: Slope, flux per oxygen concentration.

**Table A2:** POS sensor calibration in LAB and ultramarathon experiments.

	R1		J°O_2_ POS		Experiment
O2k Serial Number	A	B	A	B	
LAB					
G-0003	2.0851	2.2999	2.70	2.95	Exp. 1.1
G-0006	1.9956	2.0786	2.57	2.67	Exp. 1.2
G-0064	1.8829	1.8695	2.45	2.41	Exp. 1.3
G-0008	2.0124	2.1300	2.57	2.74	Exp. 1.4
G-0006	1.9900	2.0883	2.56	2.68	Exp. 1.5
F-0050	2.0575	1.9936	2.65	2.57	Exp. 1.6
G-0064	1.9910	2.0127	2.58	2.59	Exp. 1.7
G-0063	2.1835	1.8911	2.81	2.43	Exp. 1.8
G-0063	2.1778	1.8760	2.79	2.40	Exp. 2.1
PRE					
G-0002	1.9937	1.9171	2.57	2.47	Part. 3, 8
G-0006	2.0233	2.0927	2.60	2.69	Part. 7, 10
G-0008	2.0502	1.9348	2.64	2.49	Part. 4
G-0062	2.0845	2.3000	2.70	2.95	Part. 2, 9
G-0063	2.1757	1.9804	2.80	2.54	Part. 1
G-0064	2.0203	2.0680	2.61	2.67	Part. 5,6
POST					
G-0002	1.9926	1.9679	2.57	2.53	Part. 3, 8
G-0006	2.0280	2.0724	2.61	2.66	Part. 7
G-0008	2.0410	1.9473	2.62	2.51	Part. 4, 5
G-0062	2.0961	2.3069	2.71	2.96	Part. 2, 9
G-0063	2.2393	1.9716	2.88	2.53	Part. 1
G-0064	2.0203	2.0680	2.61	2.67	Part. 6, 10
REC					
G-0002	1.9755	1.9808	2.55	2.55	Part. 3, 8
G-0006	2.0087	2.0628	2.58	2.65	Part. 7, 10
G-0008	2.0339	1.9403	2.61	2.50	Part. 4, 5
G-0062	2.0826	2.2991	2.69	2.95	Part. 2, 9
G-0063	2.2352	1.9420	2.87	2.49	Part. 1
G-0064	2.0312	2.0677	2.63	2.67	Part. 6

POS sensor calibration for all instruments in LAB and for 6 used O2k for the ultramarathon experiments (Exp.). Sensor calibration was performed in the morning of all 3 days in every instrument and used for each experiment performed with the same O2k, shown as participants number (Part.). R1: POS-recorded signal [V]. J°O_2_ POS: O_2_ consumption by POS [pmol/s/mL)].
